# Hepatocyte Growth Factor, a Key Tumor-Promoting Factor in the Tumor Microenvironment

**DOI:** 10.3390/cancers9040035

**Published:** 2017-04-17

**Authors:** Benjamin Yaw Owusu, Robert Galemmo, James Janetka, Lidija Klampfer

**Affiliations:** 1 Department of Oncology, Drug Discovery Division, Southern Research Institute, Birmingham, AL 35205, USA; bnyowusu@uab.edu; 2 Protexase Therapeutics, St. Louis, MO 63110, USA; galemmo@protexase.com; 3 Department of Biochemistry and Molecular Biophysics, Alvin J. Siteman Cancer Center, Washington University in St. Louis, St. Louis, MO 63110, USA; janetkaj@wustl.edu

**Keywords:** HGF, tumor microenvironment, fibroblasts

## Abstract

The tumor microenvironment plays a key role in tumor development and progression. Stromal cells secrete growth factors, cytokines and extracellular matrix proteins which promote growth, survival and metastatic spread of cancer cells. Fibroblasts are the predominant constituent of the tumor stroma and Hepatocyte Growth Factor (HGF), the specific ligand for the tyrosine kinase receptor c-MET, is a major component of their secretome. Indeed, cancer-associated fibroblasts have been shown to promote growth, survival and migration of cancer cells in an HGF-dependent manner. Fibroblasts also confer resistance to anti-cancer therapy through HGF-induced epithelial mesenchymal transition (EMT) and activation of pro-survival signaling pathways such as ERK and AKT in tumor cells. Constitutive HGF/MET signaling in cancer cells is associated with increased tumor aggressiveness and predicts poor outcome in cancer patients. Due to its role in tumor progression and therapeutic resistance, both HGF and MET have emerged as valid therapeutic targets. Several inhibitors of MET and HGF are currently being tested in clinical trials. Preclinical data provide a strong indication that inhibitors of HGF/MET signaling overcome both primary and acquired resistance to EGFR, HER2, and BRAF targeting agents. These findings support the notion that co-targeting of cancer cells and stromal cells is required to prevent therapeutic resistance and to increase the overall survival rate of cancer patients. HGF dependence has emerged as a hallmark of therapeutic resistance, suggesting that inhibitors of biological activity of HGF should be included into therapeutic regimens of cancer patients.

## 1. Tumor Microenvironment

**Figure 1:**
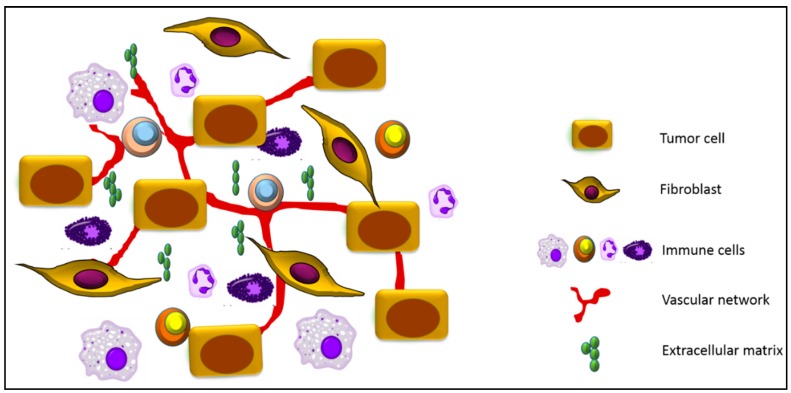
Schematic representation of the key components of the tumor microenvironment. In addition to tumor cells, the tumor niche is comprised of stromal cells such as cancer-associated fibroblasts, macrophages, lymphocytes, neutrophils and mast cells. Important components of the tumor microenvironment also include the extracellular matrix and the vascular network, which is composed of blood vessels, lymphatic vessels and pericytes.

The transition from a normal to a malignant cell is driven by progressive accumulation of mutations through which cancer cells acquire unlimited proliferative potential, resistance to apoptosis and the ability to metastasize [1,2]. Indeed, oncogene addiction, the reliance of cancer cells on oncogenic signaling for their survival, has been successfully utilized in targeted cancer therapy. However, tumors are not just a mass of malignant cells, but rather resemble abnormal organs encompassing multiple cell types, including nonmalignant cells, such as fibroblasts, immune cells, extracellular matrix (ECM), and the vascular network [3,4] (Figure 1). It has become evident that the tumor microenvironment plays a key role in tumor initiation, progression, metastasis and therapeutic resistance [5,6]. In fact, cancer cells are not only addicted to specific oncogenes, but also to pro-survival signals provided by the tumor stroma [7,8]. As such, the tumor microenvironment has emerged as an important target for therapeutic interventions.

Normal tissues have been shown to possess tumor-suppressing abilities, providing a barrier against tumorigenesis [9,10,11]. However, important changes occur during tumorigenesis, resulting in the formation of an environment that enables tumors to progress to malignancy. These alterations are driven by tumor-derived factors and involve recruitment and activation of stromal fibroblasts, polarization and education of immune cells, matrix remodeling and the development of abnormal blood vasculature [12,13,14,15,16]. In general, factors in the tumor microenvironment tend to promote tumorigenesis, however plasticity is a principal characteristic of stromal cells, and both tumor-promoting and anti-tumorigenic properties of cancer-associated fibroblasts have been described [17,18].

Secretion of a variety of cytokines, growth factors and chemokines by stromal cells generates a pro-inflammatory microenvironment, which shares characteristics with wound healing. Indeed, tumors have been viewed as wounds that fail to heal [19]. Accordingly, several anti-inflammatory drugs have chemopreventive and therapeutic activity. For example, sulindac significantly reduces the number of polyps in Familial Adenomatous Polyposis (FAP) patients who harbor a mutation in the *APC* gene [20] and the use of aspirin is associated with a better clinical outcome in colon cancer patients [21].

**Figure 2:**
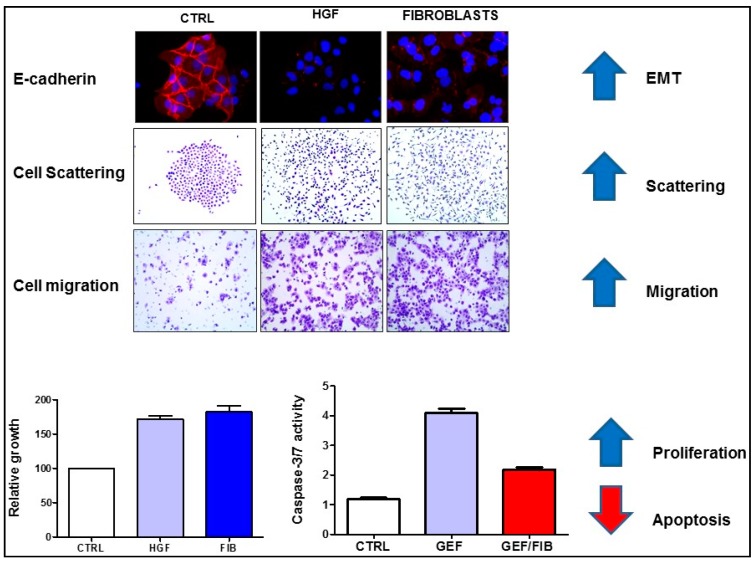
Hepatocyte growth factor (HGF) or HGF-producing fibroblasts (FIB) promote epithelial-mesenchymal transition (EMT), associated with inhibition of E-cadherin expression (red fluorescence), enhance cell scattering and migration, increase proliferation of cancer cells and confer resistance to apoptosis. The cell migration assay shows the number of cells that migrated through the membrane of a transwell chamber. Apoptosis is shown as increased caspase-3/7 activity in gefitinib (GEF)-treated colon cancer cells, which is blocked by fibroblasts. The figure is modified from our recent publication [24].

Fibroblasts and myofibroblasts are found abundant in the tumor stroma and secrete several tumor-promoting chemokines, growth factors, cytokines and extracellular matrix proteins. Hepatocyte growth factor (HGF) is a major component of the fibroblast secretome [22] and cancer-associated fibroblasts have been shown to promote epithelial-mesenchymal transition, cell scattering and migration of cancer cells in an HGF-dependent manner. In addition, fibroblasts (or recombinant HGF) promote survival of cancer cells and represent an important source of primary and acquired resistance to targeted therapy, including inhibitors of EGFR (Figure 2). Finally, myofibroblasts have been shown to promote Wnt signalling and foster cancer stem cell phenotype by promoting Wnt signaling through production of hepatocyte growth factor (HGF) [23].

Classification of colon cancer patients based on distinct global gene expression profiles has been shown to have prognostic and predictive significance [25,26,27]. According to this classification, patients with cancers characterized by the stemness/serrated/mesenchymal (SSM) gene signature have a poor prognosis. However, careful analysis of these classification systems by Calon et al. established that the predictive power of this gene signature is derived from gene expression in stromal rather than in epithelial cells [28]. The authors demonstrated that TGF-β signaling in cancer- associated fibroblasts (CAFs) increased the frequency of tumor-initiating cells, a common feature of all colorectal cancer subtypes with poor prognosis. Accordingly, pharmacological inhibition of TGF-β signaling blocked the crosstalk between cancer cells and fibroblasts and prevented metastatic spread [28]. Another group confirmed that the CAF signature was associated with poor prognosis in untreated colon cancer patients and predicted resistance to radiotherapy in rectal cancer [29]. These studies confirmed that stroma significantly contributes to clinical features of colorectal cancer and shapes the response to therapy.

Thus, it is becoming clear that drugs which would normalize the tumor stroma or would block signaling between stroma and tumor cells should be incorporated into therapeutic regimens for cancer patients in order to control cancer spread and/or to prevent cancer recurrence. Tumor cells are dynamic, and ever-evolving genetic and epigenetic changes pose a serious challenge for cancer therapy. In contrast, cells in the tumor microenvironment are genetically stable and the tumor-promoting nature of the tumor microenvironment is reversible, suggesting that the tumor microenvironment may be a preferred target for therapeutic approaches.

## 2. HGF/MET Signaling in the Tumor Microenvironment

HGF has been identified as a scattering factor for epithelial cells [30,31,32] and, independently, as a fibroblast-secreted factor that promotes the motility of epithelial cells [33]. Binding of HGF to its receptor, MET, leads to receptor dimerization and induction of signaling pathways that support growth, survival, motility and metastatic spread of cancer cells. Although HGF is the sole ligand for MET, growth factors such as EGF and TGFα have been shown to induce delayed activation of MET, which depends on the EGFR kinase activity [34]. In fact, the crosstalk between EGFR and MET maximizes the oncogenic activity of EGFR and leads to increased migration and invasion of lung cancer cells [34].

**Figure 3:**
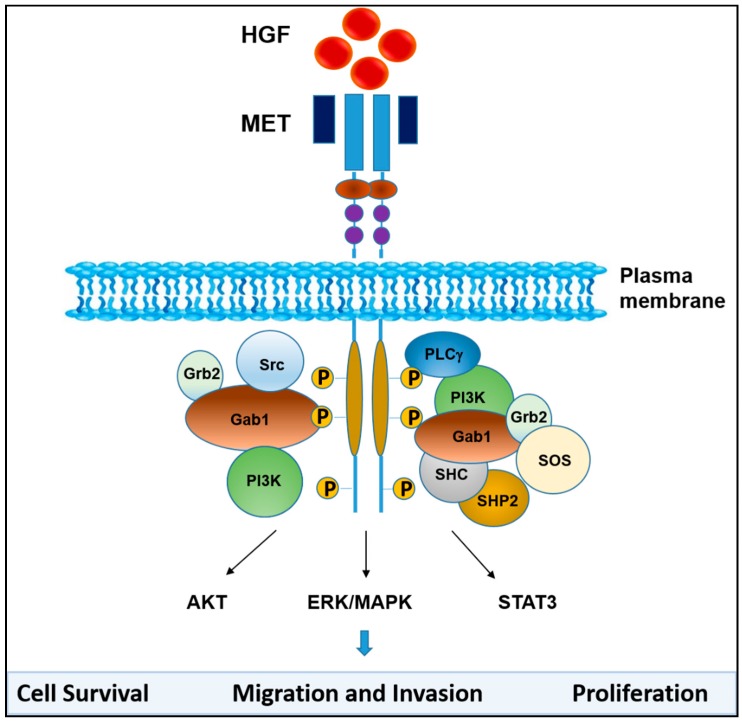
HGF/MET signaling. Binding of HGF to MET induces conformational changes that result in receptor dimerization and trans-phosphorylation of tyrosine residues in the catalytic domain of MET and phosphorylation of tyrosine residues in the carboxyl-terminal tail. The phosphorylated tyrosine residues create docking sites for several adaptor molecules and kinase substrates as indicated. MET activation leads to subsequent activation of signaling pathways that include MAPK, PI3K/AKT and STAT3, which mediate MET-dependent cell proliferation, survival, migration and invasion.

MET activation triggers Ras-dependent ERK1/ERK2 activation and STAT3 signaling, which contribute to enhanced proliferation, survival and migration of cancer cells (Figure 3). HGF-induced MET activation also triggers multiple pro-survival pathways in cancer cells, such as AKT and STAT3, promotes epithelial-mesenchymal transition (EMT), and thus confers primary and acquired resistance to anti-cancer therapy [35,36,37,38,39].

HGF/MET signaling plays a crucial role in embryogenesis, organogenesis, wound healing and tissue repair, at least in part by stimulating epithelial to mesenchymal transition (EMT). Indeed, HGF or MET deficiency in mice is embryonically lethal. However, constitutive activation of the HGF/MET signaling pathway promotes the growth and survival of cancer cells and stimulates their metastatic spread [40]. Accordingly, activation of the HGF/MET signaling pathway in tumor cells is associated with tumor aggressiveness and resistance to therapy, and predicts poor outcome in cancers patients [41]. Colon cancer patients, particularly patients with lymph node and liver metastasis, have increased levels of HGF in serum and in tumor tissues [42]. Elevated levels of HGF are associated with poor survival of stage II and stage III colon cancer patients [43]. High levels of HGF also correlate with lymph node metastasis and relapse in breast cancer patients [44,45], multiple myeloma patients [46] and myeloid leukemia patients [47].

**Figure 4:**
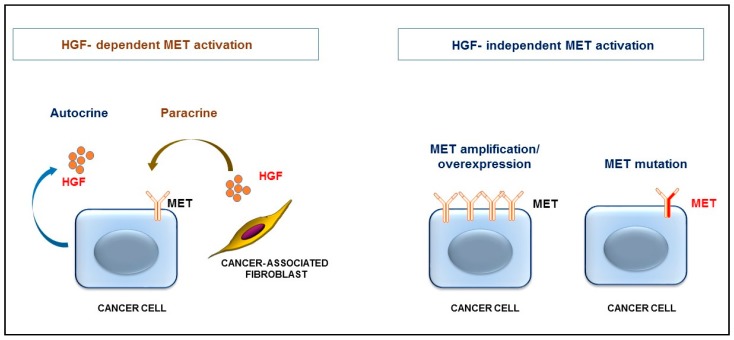
HGF-dependent and HGF-independent MET activation. Cancer cells can produce pro-HGF (due to mutations in the HGF promoter or expression of oncogenic transcription factors), which activates MET in an autocrine manner. More commonly, pro-HGF is produced by stromal cells, such as fibroblasts, and HGF activates MET in a paracrine manner. Ligand-independent MET activation occurs due to overexpression or amplification of MET or due to mutational activation of MET.

HGF has recently been shown to be constitutively produced in a relatively large subset (~30%) of primary colon tumors and in established colon cancer cell lines due to mutations in the HGF promoter region [48]. HGF-producing colon cancer cells display autocrine activation of MET signaling (Figure 4). Similar mutations in the HGF promoter region also occur in breast cancer cells [49]. Increased levels of HGF have been found in the bone marrow of acute myeloid leukemia (AML) patients [50]. About 50% of adult AML cell lines and primary specimens secrete high levels of HGF, which activates MET in an autocrine manner [51,52]. Fusion transcription factors, such as AML1/ETO and PLZ-RARα appear to be sufficient to induce the expression of pro-HGF [51]. Interestingly, HGF mutations were detected in 10.5% of lung adenocarcinomas and in 5.8% of lung squamous carcinomas [53]; however, the functional significance of these mutations is currently not understood.

In general, autocrine production of HGF by cancer cells occurs infrequently. More commonly HGF is produced by stromal cells, such as cancer-associated fibroblasts, and triggers MET activation in a paracrine fashion (Figure 4). In fact, HGF appears to be a crucial protein for the cross-talk between cancer cells and cancer-associated fibroblasts [24,54,55,56]. Targeted deletion of IKKβ in fibroblasts which increased HGF expression, triggered increased proliferation of intestinal epithelial cells and enhanced inflammation-induced tumor formation in IKKβ mutant mice [54]. Accordingly, pharmacological inhibition of MET prevented the tumor-promoting activity of IKKβ-deficient fibroblasts, confirming the significance of HGF/MET signaling for the crosstalk between cancer cells and tumor-promoting fibroblasts. Targeted deletion of epimorphin, which decreased expression of HGF in myofibroblasts, reduces polyposis in *Apc^Min/+^* mice, indicating that epimorphin exerts oncogenic potential via remodeling of the stromal microenvironment [57]. Tumor progression locus 2 (TPL2) deficiency leads to increased HGF expression in intestinal fibroblasts, coupled to increased MET activation in epithelial cells [58]. TPL2 has been recently shown to have tumor suppressor properties in the *Apc^Min/+^* model [59]. 

However, regardless of its cellular origin, HGF is always secreted as pro-HGF, an inactive precursor. While capable of binding to MET, pro-HGF does not trigger MET activation, and therefore acts as a receptor antagonist. A proteolytically inert mutant of pro-HGF confirmed the competitive antagonism between HGF and pro-HGF, and suppressed proliferation, motility and invasiveness of cancer cells in vitro and inhibited tumor growth and metastases in vivo [60]. Proteolytic conversion of pro-HGF to its active form is the rate-limiting step in the HGF/MET signaling pathway. The trypsin-like serine proteases, matriptase, hepsin and HGF activator (HGFA), which are commonly over-expressed in tumor cells, are three principal proteases responsible for HGF activation [61,62,63,64,65,66,67,68]. These enzymes cleave pro-HGF to HGF 10^2^–10^4^ times more efficiently than, for example, TMPRSS13 (Transmembrane Protease, Serine 13) or uPA (urokinase plasminogen activator) [68,69]. The activity of matriptase, HGFA and hepsin is controlled by the endogenous inhibitors of pro-HGF activation, the HGFA inhibitors (HAI)-1/2 [68,70,71], whose expression is reduced in tumor tissues. Intestinal deletion of endogenous HAI-1 augments Wnt signaling in *Apc^Min/+^* mice, both in tumors and in normal mucosa, and enhances intestinal tumor formation [72], confirming that HAI-1 has tumor suppressor properties. Accordingly, reduced expression of HAIs is associated with advanced disease and poor outcome in cancer patients [72,73,74,75,76,77,78]. Small molecule inhibitors of HGFA and antibodies neutralizing HGFA and matriptase have been developed [79] as potential therapeutic agents. However, we developed the first small molecule triplex inhibitors of HGFA, matriptase and hepsin [80,81,82], and confirmed that they block oncogenic HGF/MET signaling [24].

MET mutations, MET amplifications or MET overexpression, which trigger ligand-independent activation of MET signaling (Figure 4), are rare in primary human cancer [53]. MET activating mutations in cancer have been described in renal papillary carcinomas, hepatocellular carcinomas [83], small-cell lung cancer and colon cancers [84]. However, MET mutations are frequently detected in metastatic disease and increased expression/amplification of MET in colorectal cancer patients has been shown to promote the metastatic spread of cancer [85]. Recently it has been established that MET-positive breast cancer cells, such as triple negative breast cancer, preferentially metastasize to the brain via induction of IL-1β. MET-induced IL-1β triggers pro-HGF secretion in tumor-associated astrocytes, establishing a pro-metastatic inflammatory tumor microenvironment [86].

In addition, MET amplifications were detected in a significant number of lung- and colon cancer patients with acquired resistance to anti-EGFR therapy [87,88] (see below). While MET-amplified cancer cells do not respond to EGFR-targeting drugs, they are uniquely sensitive to anti-MET therapy.

## 3. HGF/MET Signaling Is a Hallmark of Therapeutic Resistance

Conventional cancer therapy, including chemotherapy and radiation, does not distinguish between normal and cancer cells. In contrast, targeted therapeutic agents, which block individual pathways that cancer cells are addicted to (such as EGFR, BRAF, MET or HER2 signaling), are specific for cancer cells and have potentially fewer side effects. However, tumors are extremely heterogeneous and cancer cells within a single tumor show extensive genetic, epigenetic and metabolic differences. Such differences have important consequences for the diagnosis and the targeted treatment of cancer. Factors in the tumor microenvironment, including HGF, promote tumor heterogeneity, at least in part, by providing an appropriate niche for cancer stem cells (CSC) [89,90].

Only a small population of patients respond to targeted therapy (*de novo resistance*), and patients that initially show a dramatic response to therapy develop resistance within months (*acquired resistance*). This limits the efficiency of targeted therapeutic approaches and results in local or systemic cancer recurrence. Therapeutic resistance is indeed the major cause of failure in curing cancer patients. One of the ten recommendations of the Blue Ribbon Panel to achieve the Cancer Moonshot goal was to “Identify therapeutic targets to overcome drug resistance through studies that determine the mechanisms that lead cancer cells to become resistant to previously effective treatments”.

Resistance to therapy can develop due to genetic changes in cancer cells that confer a therapy resistant phenotype. For example, mutations in the *KRAS* gene are associated with resistance to anti-EGFR drugs, which are approved selectively for colon cancer patients with wild type (WT) KRAS. In addition, the tumor microenvironment is a frequent source of resistance to therapy. HGF has been identified as a factor in the tumor microenvironment that blocks the response to cancer therapy. MET activation has been shown to underlie the resistance to drugs targeting EGFR, FGFR, BRAF, VEGF and HER2, demonstrating that MET activation is a general feature of resistance to targeted therapy [38,91]. Among proposed predictive biomarkers for HGF/MET targeting are determination of MET expression by immunohistochemistry, MET copy number changes and monitoring the levels of MET and HGF in plasma. However, none of these biomarkers have been vigorously tested in cancer patients.

How does HGF inhibit the response to therapy? HGF promotes epithelial mesenchymal transition (EMT) (Figure 2) which is likely to contribute to its ability to confer resistance to therapeutic approaches. It has been demonstrated that cells that underwent EMT are more resistant to cell death and display resistance to therapy [92,93]. Accordingly, decreased expression of E-cadherin, a hallmark of the mesenchymal phenotype, is associated with resistance to inhibitors of EGFR [94]. HGF promotes EMT by inducing the expression of EMT-associated transcription factors, including Snail1 [95] and Zeb1 [96]. We and others have demonstrated that Snail is sufficient to protect cancer cells from apoptosis [97,98,99]. Snail confers resistance to classical chemotherapy, but also to immunotherapy [100] and targeted therapy [101,102].

Finally, EMT promotes acquisition of a stem cell phenotype, generating cells that are extremely resistant to therapy. Activation of anti-apoptotic signaling pathways, such as Wnt and Notch, and proliferative/metabolic quiescence contribute to the drug-resistant phenotype of cancer stem cells (CSCs). Indeed, myofibroblast-derived HGF has been shown to induce Wnt signaling in colon cancer cells and to confer the cancer stem cell phenotype in vitro and in vivo [23]. MET activation also promotes the cancer stem cell phenotype in several other types of cancer, including gliomas [103,104], colon cancer [55], head and neck cancer [105], prostate cancer [106] and pancreatic cancer [107].

**Figure 5:**
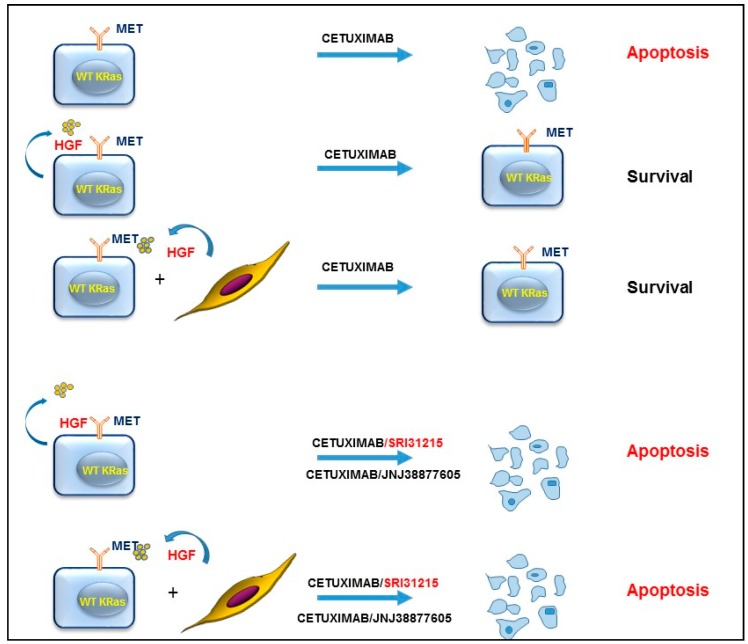
Inhibition of HGF/MET signaling overcomes resistance to inhibitors of EGFR (such as cetuximab) in colon cancer cells. Inhibition of MET by JNJ3887605 or inhibition of pro-HGF activation by SRI31215 overcomes resistance mediated by autocrine HGF/MET signaling, or fibroblast-mediated resistance to cetuximab in colon cancer cells with WT KRAS [24].

We demonstrated that HGF or HGF-producing fibroblasts conferred resistance to EGFR targeting therapy by reactivation of pro-survival pathways in cancer cells, including ERK and AKT activation [24]. Inhibition of the MET kinase activity by JNJ38877605 or inhibition of the biological activity of HGF by SRI31215, a novel small molecule inhibitor of pro-HGF activation, restored sensitivity of HGF-producing colon cancer cells to EGFR inhibition by blocking autocrine MET activation. SRI31215 or JNJ38877605 also overcame resistance mediated by HGF-producing fibroblasts, demonstrating that inhibition of HGF/MET signaling prevents tumor-microenvironment-mediated resistance to targeted therapy (Figure 5). Co-inhibition of EGFR and MET promotes eradication of colon cancer stem cells, resulting in durable tumor regression [55].

Consistent with preclinical studies, increased levels of HGF in colon cancer patients with WT KRAS, and in NSCLC patients correlate with lack of response to EGFR inhibitors [108,109].

EGFR targeting drugs have also significantly improved the outcome of lung cancer patients. Most NSCLC patients with EGFR mutations initially respond to treatment with EGFR tyrosine kinase inhibitors (TKI), but resistance develops rapidly in virtually all patients. Acquired resistance to EGFR inhibitors has been associated frequently with selection for secondary EGFR mutations, such as T790M in exon 20 [110,111], or with MET amplifications [112]. Most troublingly, MET amplification confers resistance to first- and third-generation of EGFR inhibitors.

MET-amplified lung cancers are addicted to MET signaling and are therefore extremely sensitive to MET inhibition [113]. Several MET TKI and MET specific antibodies have entered clinical trials [79,114]. However, based on preclinical data, acquired resistance to MET kinase inhibitors is likely to occur rapidly in cancer patients as well. Moreover, it has been shown that acquired resistance to kinase inhibitors, which prompts discontinuation of this therapy, is associated with accelerated disease progression. MET kinase inhibitors block MET endocytosis, resulting in an increased number of cell surface receptors and subsequent re-activation of MET signaling [115]. In some cases, a switch to EGFR dependency has been shown to underlie the resistance to MET kinase inhibitors [116,117]. However, some lung cancer cells fail to respond to combined treatment with EGFR and MET inhibitors or develop resistance to dual EGFR/MET inhibition [118]. Thus, novel therapeutic targets and rationally designed combination therapies are needed to enhance the initial response to therapy and to overcome acquired therapeutic resistance.

**Figure 6:**
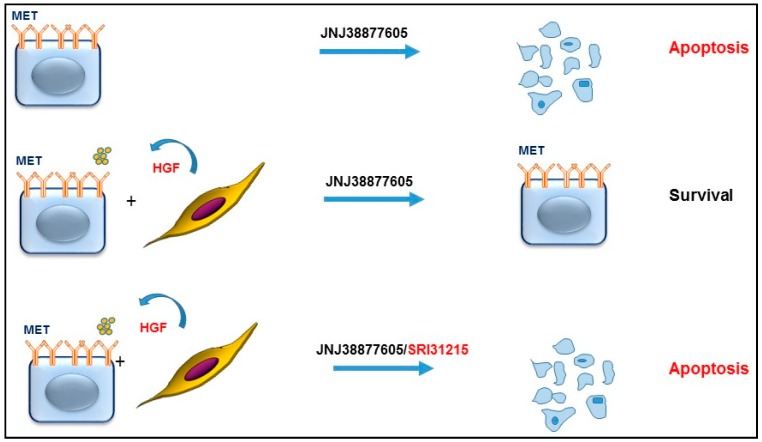
Dual inhibition of MET and HGF in MET-amplified cancer cells prevents resistance to targeted therapy. MET-amplified cancer cells are selectively sensitive to MET inhibition. However, upon pharmacological inhibition of MET (JNJ3887605), cancer cells become addicted to HGF, commonly provided by the tumor microenvironment. Thus, co-inhibition of HGF activity by SRI31215 prevents the development of resistance to MET-targeting agents.

Owusu et al. [119] and others [35] have demonstrated that HGF drives resistance to anti-MET therapy in MET-amplified lung cancer cells (Figure 6), revealing that MET-amplified NSCLC cells become addicted to HGF upon MET inhibition. HGF or pro-HGF-producing fibroblasts inhibit not only the response to individual treatment with a MET kinase inhibitor, but also the response to dual inhibition of EGFR and MET [119]. We demonstrated that HGF reactivates MET, EGFR and RON signaling and restores AKT, ERK and WNK1 activation in MET-inhibited cells. Thus, upfront inhibition of HGF and MET, or triple inhibition of EGFR, MET and HGF, may be required to prevent the development of resistance to targeted therapy in MET-amplified NSCLC cells. Supporting this notion, it has been shown that HGF is required for optimal activation of the MET kinase in MET amplified cancer cells [120].

We demonstrated that SRI31215, an inhibitor of pro-HGF activation, blocked crosstalk between tumor cells and fibroblasts in MET-amplified NSCLC cells. SRI31215 overcame fibroblast-mediated resistance to MET inhibition or to dual MET/EGFR inhibition by preventing fibroblast-mediated reactivation of AKT and ERK signaling. Structurally-unrelated triplex inhibitors of pro-HGF activation that we developed in parallel showed similar biological activity [119].

## 4. Conclusions

Constitutive HGF/MET signaling is a hallmark of cancer cells and HGF and MET have both emerged as valid therapeutic targets. Some cancer cells produce HGF which stimulates MET in an autocrine manner. More commonly, HGF is present in the tumor microenvironment and activates MET expressed on tumor cells in a paracrine fashion. HGF promotes proliferation, migration, invasion and survival of cancer cells and confers resistance to therapy. In contrast to resistance that arises due to genetic alterations, tumor-microenvironment-mediated resistance is *transient* and cancer cells regain sensitivity to therapy when isolated from the microenvironment. Thus, “normalizing” the tumor microenvironment or inhibiting communication between tumor cells and the tumor microenvironment is an important strategy to combat therapeutic resistance.

Preclinical studies offer a strong support to include inhibitors of HGF/MET signaling into therapeutic strategies. Although several MET kinase inhibitors have entered clinical trials, novel approaches for suppressing HGF/MET signaling are needed, due to resistance to kinase inhibitors. Moreover, we and others have demonstrated that inhibition of both MET and HGF is required to overcome therapeutic resistance in MET-amplified cancer cells. We developed the first small molecule inhibitors of pro-HGF activation and demonstrated that they efficiently block HGF/MET signaling and overcome HGF-mediated resistance to targeted therapy. Thus, agents blocking the biological activity of HGF, such as inhibitors of pro-HGF activation, should be incorporated into therapeutic regimens for a selected population of cancer patients.

## References

[1] Hanahan D., Weinberg R.A. (2011). Hallmarks of cancer: The next generation. Cell.

[2] Hanahan D., Weinberg R.A. (2000). The hallmarks of cancer. Cell.

[3] Egeblad M., Nakasone E.S., Werb Z. (2010). Tumors as organs: Complex tissues that interface with the entire organism. Dev. Cell.

[4] Li H., Fan X., Houghton J. (2007). Tumor microenvironment: The role of the tumor stroma in cancer. J. Cell. Biochem..

[5] Quail D.F., Joyce J.A. (2013). Microenvironmental regulation of tumor progression and metastasis. Nat. Med..

[6] Sun Y. (2016). Tumor microenvironment and cancer therapy resistance. Cancer Lett..

[7] Gascard P., Tlsty T.D. (2016). Carcinoma-associated fibroblasts: Orchestrating the composition of malignancy. Genes Dev..

[8] Lisanti M.P., Martinez-Outschoorn U.E., Sotgia F. (2013). Oncogenes induce the cancer-associated fibroblast phenotype: Metabolic symbiosis and “fibroblast addiction” are new therapeutic targets for drug discovery. Cell Cycle.

[9] Dong-Le Bourhis X., Berthois Y., Millot G., Degeorges A., Sylvi M., Martin P.M., Calvo F. (1997). Effect of stromal and epithelial cells derived from normal and tumorous breast tissue on the proliferation of human breast cancer cell lines in co-culture. Int. J. Cancer J. Int. Cancer.

[10] Zitvogel L., Tesniere A., Kroemer G. (2006). Cancer despite immunosurveillance: Immunoselection and immunosubversion. Nat. Rev. Immunol..

[11] Bissell M.J., Hines W.C. (2011). Why don’t we get more cancer? A proposed role of the microenvironment in restraining cancer progression. Nat. Med..

[12] Ruffell B., Affara N.I., Coussens L.M. (2012). Differential macrophage programming in the tumor microenvironment. Trends Immunol..

[13] Ruffell B., DeNardo D.G., Affara N.I., Coussens L.M. (2010). Lymphocytes in cancer development: Polarization towards pro-tumor immunity. Cytokine Growth Factor Rev..

[14] Egeblad M., Werb Z. (2002). New functions for the matrix metalloproteinases in cancer progression. Nat. Rev. Cancer.

[15] Gudjonsson T., Ronnov-Jessen L., Villadsen R., Rank F., Bissell M.J., Petersen O.W. (2002). Normal and tumor-derived myoepithelial cells differ in their ability to interact with luminal breast epithelial cells for polarity and basement membrane deposition. J. Cell Sci..

[16] Levental K.R., Yu H., Kass L., Lakins J.N., Egeblad M., Erler J.T., Fong S.F., Csiszar K., Giaccia A., Weninger W. (2009). Matrix crosslinking forces tumor progression by enhancing integrin signaling. Cell.

[17] Ozdemir B.C., Pentcheva-Hoang T., Carstens J.L., Zheng X., Wu C.C., Simpson T.R., Laklai H., Sugimoto H., Kahlert C., Novitskiy S.V. (2014). Depletion of carcinoma-associated fibroblasts and fibrosis induces immunosuppression and accelerates pancreas cancer with reduced survival. Cancer Cell.

[18] Rhim A.D., Oberstein P.E., Thomas D.H., Mirek E.T., Palermo C.F., Sastra S.A., Dekleva E.N., Saunders T., Becerra C.P., Tattersall I.W. (2014). Stromal elements act to restrain, rather than support, pancreatic ductal adenocarcinoma. Cancer Cell.

[19] Dvorak H.F. (1986). Tumors: Wounds that do not heal. Similarities between tumor stroma generation and wound healing. N. Eng. J. Med..

[20] Cruz-Correa M., Hylind L.M., Romans K.E., Booker S.V., Giardiello F.M. (2002). Long-term treatment with sulindac in familial adenomatous polyposis: A prospective cohort study. Gastroenterology.

[21] Ogino S., Lochhead P., Giovannucci E., Meyerhardt J.A., Fuchs C.S., Chan A.T. (2014). Discovery of colorectal cancer PIK3CA mutation as potential predictive biomarker: Power and promise of molecular pathological epidemiology. Oncogene.

[22] Kalluri R. (2016). The biology and function of fibroblasts in cancer. Nat. Rev. Cancer.

[23] Vermeulen L., De Sousa E.M.F., van der Heijden M., Cameron K., de Jong J.H., Borovski T., Tuynman J.B., Todaro M., Merz C., Rodermond H. (2010). Wnt activity defines colon cancer stem cells and is regulated by the microenvironment. Nat. Cell Biol..

[24] Owusu B.Y., Bansal N., Venukadasula P.K., Ross L.J., Messick T.E., Goel S., Galemmo R.A., Klampfer L. (2016). Inhibition of pro-HGF activation by SRI31215, a novel approach to block oncogenic HGF/MET signaling. Oncotarget.

[25] De Sousa E.M.F., Wang X., Jansen M., Fessler E., Trinh A., de Rooij L.P., de Jong J.H., de Boer O.J., van Leersum R., Bijlsma M.F. (2013). Poor-prognosis colon cancer is defined by a molecularly distinct subtype and develops from serrated precursor lesions. Nat. Med..

[26] Marisa L., de Reynies A., Duval A., Selves J., Gaub M.P., Vescovo L., Etienne-Grimaldi M.C., Schiappa R., Guenot D., Ayadi M. (2013). Gene expression classification of colon cancer into molecular subtypes: Characterization, validation, and prognostic value. PLoS Med..

[27] Sadanandam A., Lyssiotis C.A., Homicsko K., Collisson E.A., Gibb W.J., Wullschleger S., Ostos L.C., Lannon W.A., Grotzinger C., Del Rio M. (2013). A colorectal cancer classification system that associates cellular phenotype and responses to therapy. Nat. Med..

[28] Calon A., Lonardo E., Berenguer-Llergo A., Espinet E., Hernando-Momblona X., Iglesias M., Sevillano M., Palomo-Ponce S., Tauriello D.V., Byrom D. (2015). Stromal gene expression defines poor-prognosis subtypes in colorectal cancer. Nat. Genet..

[29] Isella C., Terrasi A., Bellomo S.E., Petti C., Galatola G., Muratore A., Mellano A., Senetta R., Cassenti A., Sonetto C. (2015). Stromal contribution to the colorectal cancer transcriptome. Nat. Genet..

[30] Weidner K.M., Arakaki N., Hartmann G., Vandekerckhove J., Weingart S., Rieder H., Fonatsch C., Tsubouchi H., Hishida T., Daikuhara Y. (1991). Evidence for the identity of human scatter factor and human hepatocyte growth factor. Proc. Natl. Acad. Sci. USA.

[31] Nakamura T., Nishizawa T., Hagiya M., Seki T., Shimonishi M., Sugimura A., Tashiro K., Shimizu S. (1989). Molecular cloning and expression of human hepatocyte growth factor. Nature.

[32] Naldini L., Weidner K.M., Vigna E., Gaudino G., Bardelli A., Ponzetto C., Narsimhan R.P., Hartmann G., Zarnegar R., Michalopoulos G.K. (1991). Scatter factor and hepatocyte growth factor are indistinguishable ligands for the met receptor. EMBO J..

[33] Stoker M., Gherardi E., Perryman M., Gray J. (1987). Scatter factor is a fibroblast-derived modulator of epithelial cell mobility. Nature.

[34] Dulak A.M., Gubish C.T., Stabile L.P., Henry C., Siegfried J.M. (2011). HGF-independent potentiation of EGFR action by c-MET. Oncogene.

[35] Pennacchietti S., Cazzanti M., Bertotti A., Rideout W.M., Han M., Gyuris J., Perera T., Comoglio P.M., Trusolino L., Michieli P. (2014). Microenvironment-derived HGF overcomes genetically determined sensitivity to anti-MET drugs. Cancer Res..

[36] Lesko E., Majka M. (2008). The biological role of HGF-MET axis in tumor growth and development of metastasis. Front. Biosci. J. Virtual Libr..

[37] Liska D., Chen C.T., Bachleitner-Hofmann T., Christensen J.G., Weiser M.R. (2011). HGF rescues colorectal cancer cells from EGFR inhibition via MET activation. Clin. Cancer Res..

[38] Straussman R., Morikawa T., Shee K., Barzily-Rokni M., Qian Z.R., Du J., Davis A., Mongare M.M., Gould J., Frederick D.T. (2012). Tumour micro-environment elicits innate resistance to RAF inhibitors through HGF secretion. Nature.

[39] Junttila M.R., de Sauvage F.J. (2013). Influence of tumour micro-environment heterogeneity on therapeutic response. Nature.

[40] Comoglio P.M., Giordano S., Trusolino L. (2008). Drug development of MET inhibitors: Targeting oncogene addiction and expedience. Nat. Rev. Drug Discov..

[41] Sierra J.R., Tsao M.S. (2011). C-MET as a potential therapeutic target and biomarker in cancer. Ther. Adv. Med. Oncol..

[42] Fukuura T., Miki C., Inoue T., Matsumoto K., Suzuki H. (1998). Serum hepatocyte growth factor as an index of disease status of patients with colorectal carcinoma. Br. J. Cancer.

[43] Toiyama Y., Miki C., Inoue Y., Okugawa Y., Tanaka K., Kusunoki M. (2009). Serum hepatocyte growth factor as a prognostic marker for stage II or III colorectal cancer patients. Int. J. Cancer. J. Int. Cancer.

[44] Toi M., Taniguchi T., Ueno T., Asano M., Funata N., Sekiguchi K., Iwanari H., Tominaga T. (1998). Significance of circulating hepatocyte growth factor level as a prognostic indicator in primary breast cancer. Clin. Cancer Res..

[45] Taniguchi T., Toi M., Inada K., Imazawa T., Yamamoto Y., Tominaga T. (1995). Serum concentrations of hepatocyte growth factor in breast cancer patients. Clin. Cancer Res..

[46] Seidel C., Borset M., Turesson I., Abildgaard N., Sundan A., Waage A. (1998). Elevated serum concentrations of hepatocyte growth factor in patients with multiple myeloma. The nordic myeloma study group. Blood.

[47] Verstovsek S., Kantarjian H., Estey E., Aguayo A., Giles F.J., Manshouri T., Koller C., Estrov Z., Freireich E., Keating M. (2001). Plasma hepatocyte growth factor is a prognostic factor in patients with acute myeloid leukemia but not in patients with myelodysplastic syndrome. Leukemia.

[48] Seneviratne D., Ma J., Tan X., Kwon Y.K., Muhammad E., Melhem M., DeFrances M.C., Zarnegar R. (2015). Genomic instability causes HGF gene activation in colon cancer cells, promoting their resistance to necroptosis. Gastroenterology.

[49] Ma J., DeFrances M.C., Zou C., Johnson C., Ferrell R., Zarnegar R. (2009). Somatic mutation and functional polymorphism of a novel regulatory element in the HGF gene promoter causes its aberrant expression in human breast cancer. J. Clin. Investig..

[50] Hino M., Inaba M., Goto H., Nishizawa Y., Tatsumi N., Nishino T., Morii H. (1996). Hepatocyte growth factor levels in bone marrow plasma of patients with leukaemia and its gene expression in leukaemic blast cells. Br. J. Cancer.

[51] Kentsis A., Reed C., Rice K.L., Sanda T., Rodig S.J., Tholouli E., Christie A., Valk P.J., Delwel R., Ngo V. (2012). Autocrine activation of the MET receptor tyrosine kinase in acute myeloid leukemia. Nat. Med..

[52] Reikvam H., Nepstad I., Bruserud O., Hatfield K.J. (2013). Pharmacological targeting of the PI3K/mtor pathway alters the release of angioregulatory mediators both from primary human acute myeloid leukemia cells and their neighboring stromal cells. Oncotarget.

[53] Kandoth C., McLellan M.D., Vandin F., Ye K., Niu B., Lu C., Xie M., Zhang Q., McMichael J.F., Wyczalkowski M.A. (2013). Mutational landscape and significance across 12 major cancer types. Nature.

[54] Pallangyo C.K., Ziegler P.K., Greten F.R. (2015). IKKβ acts as a tumor suppressor in cancer-associated fibroblasts during intestinal tumorigenesis. J. Exp. Med..

[55] Luraghi P., Reato G., Cipriano E., Sassi F., Orzan F., Bigatto V., De Bacco F., Menietti E., Han M., Rideout W.M. (2014). MET signaling in colon cancer stem-like cells blunts the therapeutic response to egfr inhibitors. Cancer Res..

[56] Jia C.C., Wang T.T., Liu W., Fu B.S., Hua X., Wang G.Y., Li T.J., Li X., Wu X.Y., Tai Y. (2013). Cancer-associated fibroblasts from hepatocellular carcinoma promote malignant cell proliferation by hgf secretion. PLoS ONE.

[57] Swietlicki E.A., Bala S., Lu J., Shaker A., Kularatna G., Levin M.S., Rubin D.C. (2013). Epimorphin deletion inhibits polyposis in the *APC^min/+^* mouse model of colon carcinogenesis via decreased myofibroblast HGF secretion. Am. J. Physiol. Gastrointest. Liver Physiol..

[58] Koliaraki V., Roulis M., Kollias G. (2012). TPL2 regulates intestinal myofibroblast hgf release to suppress colitis-associated tumorigenesis. J. Clin. Investig..

[59] Serebrennikova O.B., Tsatsanis C., Mao C., Gounaris E., Ren W., Siracusa L.D., Eliopoulos A.G., Khazaie K., Tsichlis P.N. (2012). TPL2 ablation promotes intestinal inflammation and tumorigenesis in apcmin mice by inhibiting IL-10 secretion and regulatory T-cell generation. Proc. Natl. Acad. Sci. USA.

[60] Mazzone M., Basilico C., Cavassa S., Pennacchietti S., Risio M., Naldini L., Comoglio P.M., Michieli P. (2004). An uncleavable form of pro-scatter factor suppresses tumor growth and dissemination in mice. J. Clin. Investig..

[61] Forbs D., Thiel S., Stella M.C., Sturzebecher A., Schweinitz A., Steinmetzer T., Sturzebecher J., Uhland K. (2005). In vitro inhibition of matriptase prevents invasive growth of cell lines of prostate and colon carcinoma. Int. J. Oncol..

[62] Herter S., Piper D.E., Aaron W., Gabriele T., Cutler G., Cao P., Bhatt A.S., Choe Y., Craik C.S., Walker N. (2005). Hepatocyte growth factor is a preferred in vitro substrate for human hepsin, a membrane-anchored serine protease implicated in prostate and ovarian cancers. Biochem. J..

[63] Kataoka H., Hamasuna R., Itoh H., Kitamura N., Koono M. (2000). Activation of hepatocyte growth factor/scatter factor in colorectal carcinoma. Cancer Res..

[64] Lee S.L., Dickson R.B., Lin C.Y. (2000). Activation of hepatocyte growth factor and urokinase/plasminogen activator by matriptase, an epithelial membrane serine protease. J. Biol. Chem..

[65] Owen K.A., Qiu D., Alves J., Schumacher A.M., Kilpatrick L.M., Li J., Harris J.L., Ellis V. (2010). Pericellular activation of hepatocyte growth factor by the transmembrane serine proteases matriptase and hepsin, but not by the membrane-associated protease UPA. Biochem. J..

[66] Parr C., Watkins G., Mansel R.E., Jiang W.G. (2004). The hepatocyte growth factor regulatory factors in human breast cancer. Clin. Cancer Res..

[67] Szabo R., Rasmussen A.L., Moyer A.B., Kosa P., Schafer J.M., Molinolo A.A., Gutkind J.S., Bugge T.H. (2011). c-MET-induced epithelial carcinogenesis is initiated by the serine protease matriptase. Oncogene.

[68] Kawaguchi M., Kataoka H. (2014). Mechanisms of hepatocyte growth factor activation in cancer tissues. Cancers.

[69] Naldini L., Vigna E., Bardelli A., Follenzi A., Galimi F., Comoglio P.M. (1995). Biological activation of pro-HGF (hepatocyte growth factor) by urokinase is controlled by a stoichiometric reaction. J. Biol. Chem..

[70] Ye J., Kawaguchi M., Haruyama Y., Kanemaru A., Fukushima T., Yamamoto K., Lin C.Y., Kataoka H. (2014). Loss of hepatocyte growth factor activator inhibitor type 1 participates in metastatic spreading of human pancreatic cancer cells in a mouse orthotopic transplantation model. Cancer Sci..

[71] Kawaguchi M., Takeda N., Hoshiko S., Yorita K., Baba T., Sawaguchi A., Nezu Y., Yoshikawa T., Fukushima T., Kataoka H. (2011). Membrane-bound serine protease inhibitor HAI-1 is required for maintenance of intestinal epithelial integrity. Am. J. Pathol..

[72] Hoshiko S., Kawaguchi M., Fukushima T., Haruyama Y., Yorita K., Tanaka H., Seiki M., Inatsu H., Kitamura K., Kataoka H. (2013). Hepatocyte growth factor activator inhibitor type 1 is a suppressor of intestinal tumorigenesis. Cancer Res..

[73] Saleem M., Adhami V.M., Zhong W., Longley B.J., Lin C.Y., Dickson R.B., Reagan-Shaw S., Jarrard D.F., Mukhtar H. (2006). A novel biomarker for staging human prostate adenocarcinoma: Overexpression of matriptase with concomitant loss of its inhibitor, hepatocyte growth factor activator inhibitor-1. Cancer Epidemiol. Biomark. Prev..

[74] Oberst M.D., Johnson M.D., Dickson R.B., Lin C.Y., Singh B., Stewart M., Williams A., Al-Nafussi A., Smyth J.F., Gabra H. (2002). Expression of the serine protease matriptase and its inhibitor HAI-1 in epithelial ovarian cancer: Correlation with clinical outcome and tumor clinicopathological parameters. Clin. Cancer Res..

[75] Zeng L., Cao J., Zhang X. (2005). Expression of serine protease SNC19/matriptase and its inhibitor hepatocyte growth factor activator inhibitor type 1 in normal and malignant tissues of gastrointestinal tract. World J. Gastroenterol. WJG.

[76] Nakamura K., Abarzua F., Kodama J., Hongo A., Nasu Y., Kumon H., Hiramatsu Y. (2009). Expression of hepatocyte growth factor activator inhibitors (HAI-1 and HAI-2) in ovarian cancer. Int. J. Oncol..

[77] Hamasuna R., Kataoka H., Meng J.Y., Itoh H., Moriyama T., Wakisaka S., Koono M. (2001). Reduced expression of hepatocyte growth factor activator inhibitor type-2/placental bikunin (HAI-2/pb) in human glioblastomas: Implication for anti-invasive role of HAI-2/pb in glioblastoma cells. Int. J. Cancer.

[78] Morris M.R., Gentle D., Abdulrahman M., Maina E.N., Gupta K., Banks R.E., Wiesener M.S., Kishida T., Yao M., Teh B. (2005). Tumor suppressor activity and epigenetic inactivation of hepatocyte growth factor activator inhibitor type 2/SPINT2 in papillary and clear cell renal cell carcinoma. Cancer Res..

[79] Gherardi E., Birchmeier W., Birchmeier C., Vande Woude G. (2012). Targeting met in cancer: Rationale and progress. Nat. Rev. Cancer.

[80] Han Z., Harris P.K., Karmakar P., Kim T., Owusu B.Y., Wildman S.A., Klampfer L., Janetka J.W. (2016). Alpha-ketobenzothiazole serine protease inhibitors of aberrant HGF/c-MET and MSP/RON kinase pathway signaling in cancer. ChemMedChem.

[81] Venukadasula P.K., Owusu B.Y., Bansal N., Ross L.J., Hobrath J.V., Bao D., Truss J.W., Stackhouse M., Messick T.E., Klampfer L. (2016). Design and synthesis of nonpeptide inhibitors of hepatocyte growth factor activation. ACS Med. Chem. Lett..

[82] Han Z., Harris P.K., Jones D.E., Chugani R., Kim T., Agarwal M., Shen W., Wildman S.A., Janetka J.W. (2014). Inhibitors of HGFA, matriptase, and hepsin serine proteases: A nonkinase strategy to block cell signaling in cancer. ACS Med. Chem. Lett..

[83] Schmidt L., Duh F.M., Chen F., Kishida T., Glenn G., Choyke P., Scherer S.W., Zhuang Z., Lubensky I., Dean M. (1997). Germline and somatic mutations in the tyrosine kinase domain of the met proto-oncogene in papillary renal carcinomas. Nat. Genet..

[84] Neklason D.W., Done M.W., Sargent N.R., Schwartz A.G., Anton-Culver H., Griffin C.A., Ahnen D.J., Schildkraut J.M., Tomlinson G.E., Strong L.C. (2011). Activating mutation in MET oncogene in familial colorectal cancer. BMC Cancer.

[85] Di Renzo M.F., Olivero M., Giacomini A., Porte H., Chastre E., Mirossay L., Nordlinger B., Bretti S., Bottardi S., Giordano S. (1995). Overexpression and amplification of the MET/HGF receptor gene during the progression of colorectal cancer. Clin. Cancer Res..

[86] Xing F., Liu Y., Sharma S., Wu K., Chan M.D., Lo H.W., Carpenter R.L., Metheny-Barlow L.J., Zhou X., Qasem S.A. (2016). Activation of the c-MET pathway mobilizes an inflammatory network in the brain microenvironment to promote brain metastasis of breast cancer. Cancer Res..

[87] Bardelli A., Corso S., Bertotti A., Hobor S., Valtorta E., Siravegna G., Sartore-Bianchi A., Scala E., Cassingena A., Zecchin D. (2013). Amplification of the met receptor drives resistance to anti-EGFR therapies in colorectal cancer. Cancer Discov..

[88] Turke A.B., Zejnullahu K., Wu Y.L., Song Y., Dias-Santagata D., Lifshits E., Toschi L., Rogers A., Mok T., Sequist L. (2010). Preexistence and clonal selection of MET amplification in egfr mutant NSCLC. Cancer Cell.

[89] Alizadeh A.A., Aranda V., Bardelli A., Blanpain C., Bock C., Borowski C., Caldas C., Califano A., Doherty M., Elsner M. (2015). Toward understanding and exploiting tumor heterogeneity. Nat. Med..

[90] Campbell L.L., Polyak K. (2007). Breast tumor heterogeneity: Cancer stem cells or clonal evolution?. Cell Cycle.

[91] Wilson T.R., Fridlyand J., Yan Y., Penuel E., Burton L., Chan E., Peng J., Lin E., Wang Y., Sosman J. (2012). Widespread potential for growth-factor-driven resistance to anticancer kinase inhibitors. Nature.

[92] Smith B.N., Bhowmick N.A. (2016). Role of EMT in metastasis and therapy resistance. J. Clin. Med..

[93] Huang J., Li H., Ren G. (2015). Epithelial-mesenchymal transition and drug resistance in breast cancer (review). Int. J. Oncol..

[94] Witta S.E., Gemmill R.M., Hirsch F.R., Coldren C.D., Hedman K., Ravdel L., Helfrich B., Dziadziuszko R., Chan D.C., Sugita M. (2006). Restoring e-cadherin expression increases sensitivity to epidermal growth factor receptor inhibitors in lung cancer cell lines. Cancer Res..

[95] Grotegut S., von Schweinitz D., Christofori G., Lehembre F. (2006). Hepatocyte growth factor induces cell scattering through MAPK/EGR-1-mediated upregulation of snail. EMBO J..

[96] Han Y., Luo Y., Wang Y., Chen Y., Li M., Jiang Y. (2016). Hepatocyte growth factor increases the invasive potential of pc-3 human prostate cancer cells via an ERK/MAPK and ZEB-1 signaling pathway. Oncol. Lett..

[97] Vega S., Morales A.V., Ocana O.H., Valdes F., Fabregat I., Nieto M.A. (2004). Snail blocks the cell cycle and confers resistance to cell death. Genes Dev..

[98] Kaler P., Augenlicht L., Klampfer L. (2012). Activating mutations in beta-catenin in colon cancer cells alter their interaction with macrophages; the role of snail. PLoS ONE.

[99] Kaler P., Galea V., Augenlicht L., Klampfer L. (2010). Tumor associated macrophages protect colon cancer cells from trail-induced apoptosis through IL-1beta-dependent stabilization of snail in tumor cells. PLoS ONE.

[100] Kudo-Saito C., Shirako H., Takeuchi T., Kawakami Y. (2009). Cancer metastasis is accelerated through immunosuppression during snail-induced emt of cancer cells. Cancer Cell.

[101] Jiang Y., Zhao X., Xiao Q., Liu Q., Ding K., Yu F., Zhang R., Zhu T., Ge G. (2014). Snail and slug mediate tamoxifen resistance in breast cancer cells through activation of EGFR-ERK independent of epithelial-mesenchymal transition. J. Mol. Cell Biol..

[102] Ware K.E., Somarelli J.A., Schaeffer D., Li J., Zhang T., Park S., Patierno S.R., Freedman J., Foo W.C., Garcia-Blanco M.A. (2016). Snail promotes resistance to enzalutamide through regulation of androgen receptor activity in prostate cancer. Oncotarget.

[103] De Bacco F., D’Ambrosio A., Casanova E., Orzan F., Neggia R., Albano R., Verginelli F., Cominelli M., Poliani P.L., Luraghi P. (2016). Met inhibition overcomes radiation resistance of glioblastoma stem-like cells. EMBO Mol. Mead..

[104] Boccaccio C., Comoglio P.M. (2013). The met oncogene in glioblastoma stem cells: Implications as a diagnostic marker and a therapeutic target. Cancer Res..

[105] Sun S., Liu S., Duan S.Z., Zhang L., Zhou H., Hu Y., Zhou X., Shi C., Zhou R., Zhang Z. (2014). Targeting the c-MET/FZD8 signaling axis eliminates patient-derived cancer stem-like cells in head and neck squamous carcinomas. Cancer Res..

[106] Van Leenders G.J., Sookhlall R., Teubel W.J., de Ridder C.M., Reneman S., Sacchetti A., Vissers K.J., van Weerden W., Jenster G. (2011). Activation of c-MET induces a stem-like phenotype in human prostate cancer. PLoS ONE.

[107] Li C., Wu J.J., Hynes M., Dosch J., Sarkar B., Welling T.H., Pasca di Magliano M., Simeone D.M. (2011). C-MET is a marker of pancreatic cancer stem cells and therapeutic target. Gastroenterology.

[108] Takahashi N., Yamada Y., Furuta K., Honma Y., Iwasa S., Takashima A., Kato K., Hamaguchi T., Shimada Y. (2014). Serum levels of hepatocyte growth factor and epiregulin are associated with the prognosis on anti-EGFR antibody treatment in KRAS wild-type metastatic colorectal cancer. Br. J. Cancer.

[109] Kasahara K., Arao T., Sakai K., Matsumoto K., Sakai A., Kimura H., Sone T., Horiike A., Nishio M., Ohira T. (2010). Impact of serum hepatocyte growth factor on treatment response to epidermal growth factor receptor tyrosine kinase inhibitors in patients with non-small cell lung adenocarcinoma. Clin. Cancer Res..

[110] Kobayashi S., Boggon T.J., Dayaram T., Janne P.A., Kocher O., Meyerson M., Johnson B.E., Eck M.J., Tenen D.G., Halmos B. (2005). EGFR mutation and resistance of non-small-cell lung cancer to gefitinib. N. Engl. J. Med..

[111] Pao W., Miller V.A., Politi K.A., Riely G.J., Somwar R., Zakowski M.F., Kris M.G., Varmus H. (2005). Acquired resistance of lung adenocarcinomas to gefitinib or erlotinib is associated with a second mutation in the EGFR kinase domain. PLoS Med..

[112] Bean J., Brennan C., Shih J.Y., Riely G., Viale A., Wang L., Chitale D., Motoi N., Szoke J., Broderick S. (2007). Met amplification occurs with or without T790M mutations in EGFR mutant lung tumors with acquired resistance to gefitinib or erlotinib. Proc. Natl. Acad. Sci. USA.

[113] Smolen G.A., Sordella R., Muir B., Mohapatra G., Barmettler A., Archibald H., Kim W.J., Okimoto R.A., Bell D.W., Sgroi D.C. (2006). Amplification of met may identify a subset of cancers with extreme sensitivity to the selective tyrosine kinase inhibitor PHA-665752. Proc. Natl. Acad. Sci. USA.

[114] Ye S., Li J., Hao K., Yan J., Zhou H. (2016). The efficacy and risk profile of c-MET inhibitors in Non-small Cell Lung Cancer: A meta-analysis. Sci. Rep..

[115] Pupo E., Ducano N., Lupo B., Vigna E., Avanzato D., Perera T., Trusolino L., Lanzetti L., Comoglio P.M. (2016). Rebound effects caused by withdrawal of met kinase inhibitor are quenched by a met therapeutic antibody. Cancer Res..

[116] McDermott U., Pusapati R.V., Christensen J.G., Gray N.S., Settleman J. (2010). Acquired resistance of non-small cell lung cancer cells to MET kinase inhibition is mediated by a switch to epidermal growth factor receptor dependency. Cancer Res..

[117] Zhang Y.W., Staal B., Essenburg C., Lewis S., Kaufman D., Vande Woude G.F. (2013). Strengthening context-dependent anticancer effects on non-small cell lung carcinoma by inhibition of both MET and EGFR. Mol. Cancer Ther..

[118] Yamaoka T., Ohmori T., Ohba M., Arata S., Kishino Y., Murata Y., Kusumoto S., Ishida H., Shirai T., Hirose T. (2016). Acquired resistance mechanisms to combination MET-TKI/EGFR-TKI exposure in MET-amplified EGFR-TKI resistant lung adenocarcinoma harboring an activating EGFR mutation. Mol. Cancer Ther..

[119] Owusu B.Y., Thomas S., Venukadasula P., Han Z., Janetka J., Galemmo R., Klampfer L. (2017). Targeting the tumor-promoting microenvironment in met-amplified NSCLC cells with a novel inhibitor of pro-HGF activation. Oncotarget.

[120] Michieli P., Basilico C., Pennacchietti S., Maffe A., Tamagnone L., Giordano S., Bardelli A., Comoglio P.M. (1999). Mutant MET-mediated transformation is ligand-dependent and can be inhibited by HGF antagonists. Oncogene.

